# *Lactobacillus acidophilus* DDS-1 Modulates the Gut Microbial Co-Occurrence Networks in Aging Mice

**DOI:** 10.3390/nu14050977

**Published:** 2022-02-25

**Authors:** Ravichandra Vemuri, Christopher J. Martoni, Kylie Kavanagh, Rajaraman Eri

**Affiliations:** 1 Department of Pathology, Section on Comparative Medicine, Wake Forest School of Medicine, Medical Center Boulevard, Winston-Salem, NC 27157, USA; kkavanag@wakehealth.edu; 2 College of Health and Medicine, School of Health Sciences, University of Tasmania, Launceston, TAS 7248, Australia; rajaraman.eri@utas.edu.au; 3 Chr. Hansen, Windsor, WI 53598, USA; cachma@chr-hansen.com; 4 Department of Biomedicine, University of Tasmania, Hobart, TAS 7000, Australia

**Keywords:** aging, intestinal microbiota, dysbiosis, probiotics, microbial co-occurrences

## Abstract

Age-related alterations in the gut microbiome composition and its impacts on the host’s health have been well-described; however, detailed analyses of the gut microbial structure defining ecological microbe–microbe interactions are limited. One of the ways to determine these interactions is by understanding microbial co-occurrence patterns. We previously showed promising abilities of *Lactobacillus acidophilus* DDS-1 on the aging gut microbiome and immune system. However, the potential of the DDS-1 strain to modulate microbial co-occurrence patterns is unknown. Hence, we aimed to investigate the ability of *L. acidophilus* DDS-1 to modulate the fecal-, mucosal-, and cecal-related microbial co-occurrence networks in young and aging C57BL/6J mice. Our Kendall’s tau correlation measures of co-occurrence revealed age-related changes in the gut microbiome, which were characterized by a reduced number of nodes and associations across sample types when compared to younger mice. After four-week supplementation, *L. acidophilus* DDS-1 differentially modulated the overall microbial community structure in fecal and mucosal samples as compared to cecal samples. Beneficial bacteria such as *Lactobacillus, Oscillospira,* and *Akkermansia* acted as connectors in aging networks in response to *L. acidophilus* DDS-1 supplementation. Our findings provided the first evidence of the DDS-1-induced gut microbial ecological interactions, revealing the complex structure of microbial ecosystems with age.

## 1. Introduction

The gut microbiome is a complex but relatively stable community comprising a diverse range of microbes [1]. The interactions between these microbes and the host are an important factor in defining host health [2]. These interactions enable our microbiome to prime our immune system, influence host metabolism, and improve health by providing important metabolites such as short-chain fatty acids (SCFAs) [3]. Alterations in microbiome or dysbiosis have been identified in various diseases and physiologies, including changes with advancing age [4,5,6,7].

Multiple studies have demonstrated differences in microbial populations associated with specific regions of the gastrointestinal tract (GIT) [8,9,10]. Additionally, studies have shown intestinal location-specific dysbiosis when compared to the fecal microbiome with age and immunologic diseases in animals and human studies [6,11,12]. While the sampling of GIT is quite challenging, most of the host–microbe interactions occur at intestinal mucosal sites [11,12]. These interactions are relevant as the mucosal microbiome plays an important role in innate immunity [6,13]. Linking the microbial patterns which drive microbial compositions remains a central goal in understanding gut microbial ecology [14]. Identifying the determinant of niche-specific microbial networks requires looking beyond taxonomic, sample-level comparisons [15]. For example, in the colon, there are up to 10^12^ microbes per gram of luminal content, and due to the higher bacterial load, there may be competition between colonic resident microbes for nutrients and survival [16,17,18]. These microbe–microbe interactions may impact the stability and define the overall microbial community. Microbial co-occurrence patterns provide insights into complex microbial communities by disentangling the interactions between the microbes (i.e., co-occurrence or co-exclusion), thereby delineating the underlying ecological processes [19,20]. This emerging microbial co-occurrence network analysis was found to provide insightful information in soil microbial environment studies [21,22] as well as animal [16] and human studies [21,23,24]. These approaches yield a holistic view of microbial interactions occurring in a given environment. For example, by finding specific co-occurrence in a complex aged gut microbiome, we can identify the microorganisms that have the largest influence on the community regardless of their abundance. Similarly, we recently demonstrated novel microbial co-occurrence patterns, which revealed the presence of high-level networks between microbes, and these networks declined in older nonhuman primates (NHPs) [16]. The analysis showed that the overall community structure depends on the presence or absence of microbial taxa.

Probiotics are defined as, “Live microorganisms that, when administered in adequate amounts, confer a health benefit on the host” [4]. We previously demonstrated the potential of a clinically documented probiotic strain, *Lactobacillus acidophilus* DDS-1, in modulating the fecal microbiome [25], and cecal- and mucosal-associated microbiome along with metabolic profiles in aging mice as compared to their younger counterparts [23,24,26]. However, the group-wise comparisons of fecal, mucosal, and cecal microbiomes between young and aging mice, and their co-occurrence analysis to understand the overall microbiome structure, were not investigated. Therefore, we aimed to investigate the microbial co-occurrences network analysis with and without *L. acidophilus* DDS-1 probiotic supplementation in young and aging mice.

In order to explore the age-related overall community structure of the gut microbiome, we applied microbial co-occurrence analysis to fecal, mucosal, and cecal samples, whose individual compositions with and without *L. acidophilus* DDS-1 supplementation were reported in previous studies [25,26]. In the present work, by determining the age-related co-occurrence patterns, we have provided additional evidence of *L. acidophilus* DDS-1 probiotic-induced modulations in the gut microbial community assembly.

## 2. Methods

### 2.1. Probiotics

The bacterial strain utilized in the study, *L. acidophilus* DDS-1, was obtained in free-flowing lyophilized format from UAS Labs (now Chr. Hansen), Windsor, WI, USA. *L. acidophilus* DDS-1 was incorporated and administered as probiotic chow, as described [26,27].

### 2.2. Animals

A total of 32 C57BL/6J mice of both sexes, which included young mice (n = 16, 3–4 weeks, average weight = 19 g) and aging mice (n = 16, 40–41 weeks old, average weight = 25 g), were obtained from the University of Tasmania animal breeding facility and housed at 21–22 °C, with a 12 h light/dark cycle. All the mice had access to water available ad libitum and to radiation-sterilized rodent feed (Barastoc Rat and Mouse product number: 102108, Ridley Agriproducts, Melbourne, Australia). The nutritional composition is listed in Table S1. The details related to demographic and health characteristics such as body weights, age, and colonic histopathology were previously published [25,26]. All animal procedures were approved by the Animal Ethics Committee of the University of Tasmania, Australia (ethics approval number: A0015840 and approval date: 1 September 2018).

### 2.3. Design and Treatment

**Figure 1:**
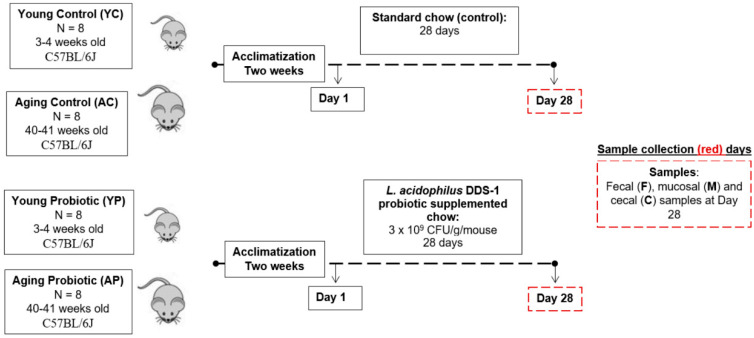
Experimental design to analyze the efficacy of *L. acidophilus* DDS-1 in young and aging C57BL/6J mice. The controls groups (YC and AC; n = 8 per group) were fed standard chow, and the treatment groups (YP and AP; n = 8 per group) were fed with chow supplemented with *L. acidophilus* DDS-1 daily for 28 days. (YC) Young control group, (YP) young probiotic group, (AC) aging control group, and (AP) aging probiotic group.

After two weeks of acclimatization, mice were randomly allocated into the following groups based on their age and treatments (n = 8 per group): (1) young control (YC), (2) young probiotic (YP), (3) aging control (AC), and (4) aging probiotic (AP). Figure 1 describes the study design and sample collection, also reported previously [25,26]. Briefly, mice in YC and AC groups received 4 g of standard mice chow mash (i.e., chow pellet blended in distilled water). The YP and AP groups received 4 g of chow mash supplemented with *L. acidophilus* DDS-1 (3 × 10 ^9^ CFU/g/mouse), freshly prepared daily for 28 days. All mice were single-caged and daily food intake was recorded. There were no differences in the daily intake of the treatment-supplemented chow among groups. All animals were euthanized on day 28.

### 2.4. Sample Collections

Fecal samples were collected on day 28. Colons were excised in a longitudinal axis from cecum to anus and their content was collected using the scraping method [26,27]. Briefly, the cecum was removed from the colon and the cecum was dissected in the longitudinal axis. Cecal content was collected by sterilized pipette tips using the scraping method, and for the mucosal content, the colon was dissected in a longitudinal axis and the content was collected utilizing a sterilized pipette tip. The colonic-mucosa were carefully collected in at least two sets from each mouse and immediately transferred into a sterile microcentrifuge tube. These samples were immediately stored at −80 °C for subsequent 16S rRNA gene sequencing and metabolomic analysis. All samples were stored at −8 °C for further analysis.

### 2.5. 16S rRNA Sequencing and Microbiome Data Analysis

The total DNA was isolated from fecal (n = 8/group), mucosal (n = 8/group), and cecal (n = 6/group) contents using the QIAamp DNA Stool Mini Kit (Qiagen, Melbourne, VIC, Australia). A high-throughput, 16S rRNA gene sequencing (V3–V4 region) was performed at the Australian Genome Research Facility (University of Queensland, Brisbane, QLD, Australia) using the Illumina MiSeq platform. The resultant data were obtained and analyzed as described previously and the sequence data have been deposited publicly in the Figshare database (DOI: https://10.6084/m9.figshare.17019947 accessed on 8 February 2022) [25,26].

### 2.6. Gram Phenotype and Microbial Co-Occurrence Analyses

To obtain functional phenotype characteristics such as the Gram-staining profile, we used METAGENassist. A bar chart was generated with percentages for each phenotypic trait associated with the individual taxon, as described previously [16]. Microbial interactions form biological networks such as microbial co-occurrence networks, which shape the structure and function of microbial communities. Microbial co-occurrence networks in specific environments have been widely developed to explore the complex gut microbial systems. To identify the overall community networks and the ecological interactions between each microbe, we performed co-occurrence analysis, and the resultant data were mapped into the microbial co-occurrence network plots using Kendall’s tau coefficient correlation, as described previously [16]. We illustrated networks of co-occurring microorganisms within communities, where microbial taxa represent nodes and the presence of a co-occurrence relationship based on correlation is represented by an edge. Each node is a microbial taxon, and the interconnecting lines are the edges. The size of the node denotes (1–9) the strength of association, while the color of the edge represents the nature of the association, such as co-occurrence (green, positive) or co-exclusion (red, negative). Kendall’s tau coefficient correlation method (more robust to outliers) uses the following formula to calculate the associations: (C−D)/(C+D), where C is the number of concordant pairs, and D is the number of discordant pairs [28]. The tau correlation coefficient returns a value ranging from −1 to 1, where all positive values represent co-occurrences and negative values represent co-exclusion. The overall network threshold with significance > 0.05 and the edge threshold of 70% were selected to control the false positive rate.

### 2.7. Multivariate and Statistical Analyses

Alpha diversity was analyzed using Shannon and Simpson’s index. Beta diversity profiles were generated using principal coordinate analysis (PCoA) using the permutational multivariate analysis of variance (PERMANOVA) test and unweighted UniFrac β diversity metrics using MEGAN 6. All data comparisons were corrected for false discovery rates. The statistical analysis was performed using GraphPad Prism software version 10 (GraphPad Software Inc., San Diego, CA, USA) for Windows with one-way analysis of variance (ANOVA), followed by Tukey’s post-hoc test for multiple comparisons and corrected for false discovery rates (*q*-values < 0.05), with a statistical significance of *p* < 0.05. To identify differential abundant microbials, linear discriminant analysis effect size (LEfSe) analyses were performed (α = 0.05), and the LDA score threshold was set 2.0 and corrected for false discovery rate.

## 3. Results

### 3.1. Animal Health Characteristics

All health characteristics for the C57BL/6J mice evaluated in this study have been previously reported [25,26]. There were no significant differences in body weight when comparing the *L. acidophilus* DDS-1-supplemented mice with controls across ages.

### 3.2. L. acidophilus DDS-1-Induced Alpha, Beta Diversity, and Taxonomic Profile Changes in Fecal, Mucosal, and Cecal Microbiomes

**Figure 2:**
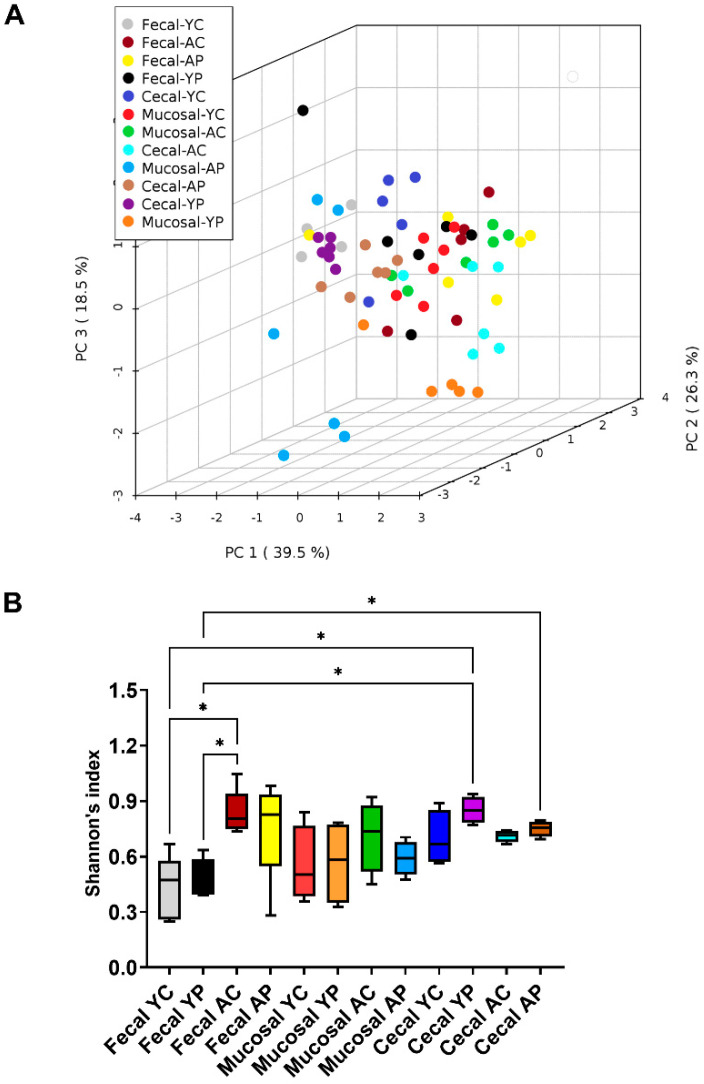
Fecal, mucosal, and cecal microbial diversity profiles in young and aging mice. (**A**) Principal coordinate analysis (PCoA) (unweighted UniFrac) showed divergence in fecal, mucosal, and cecal samples across ages (R^2^ = 0.265; *p* < 0.001). (**B**) Boxplots of pairwise comparison showing no probiotic treatment-related differences in alpha diversity profiles between the samples by Shannon’s index. * Significant differences with *p* < 0.05. The values are shown as means ± SEM. (YC) Young control group, (YP) young probiotic group, (AC) aging control group, and (AP) aging probiotic group.

In order to understand the site-specific microbiome changes, we have performed alpha, beta, and taxonomic profiling. The beta diversity profile obtained by PCoA of unweighted UniFrac distance showed significant separation between the 12 groups (*p* < 0.001) (Figure 2A). Alpha diversities obtained by Shannon’s index showed no significant differences between the control and treatment groups (Figure 2B). Individual group differences in alpha and beta diversity profiles in the fecal (F), mucosal (M), and cecal (C) microbiomes were reported previously [25,26].

**Figure 3:**
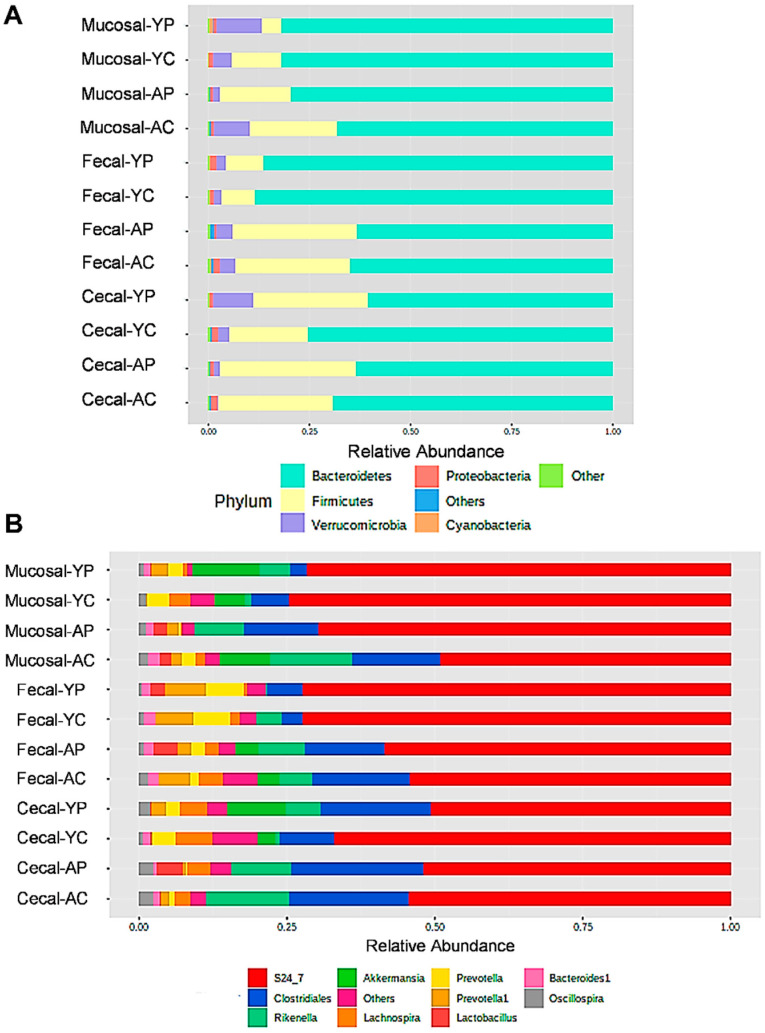
Fecal, mucosal, and cecal microbiota composition profiles in young and aging mice at the phylum (**A**) and genus levels (**B**) in control and probiotic-treated groups, revealed by 16S rRNA gene sequencing (each color represents bacterial phylum and/or genus). (YC) Young control group, (YP) young probiotic group, (AC) aging control group, and (AP) aging probiotic group.

The dominant phyla among all the groups were Firmicutes, Bacteroidetes, Verrucomicrobia, and Proteobacteria (Figure 3A). The phylum-level LEfSe analysis with significant (*p* < 0.05) phyla between groups is shown in Figure S1A. Significant increases in Proteobacteria phylum in the AC group was a noteworthy finding. At the genus level, we found that *S-24-7*, Clostridiales, *Rikenella*, and *Akkermansia* were the most dominant, as revealed by LEfSe analysis (Figure S1B). *Lactobacillus* was mostly absent in all the control groups, except in M-AC (Figure 3B) (*p* < 0.05). *L. acidophilus* DDS-1 was shown to increase *Lactobacillus* levels (*p* < 0.05) in the C-AP groups, primarily in the F-AP and C-AP groups. *Akkermansia* (Verrucomicrobia phylum) levels were upregulated in M-YP group (Figure S1B) (*p* < 0.05).

### 3.3. L. acidophilus DDS-1 Effect on Gram-Negative Bacteria in Aging Mice

**Figure 4:**
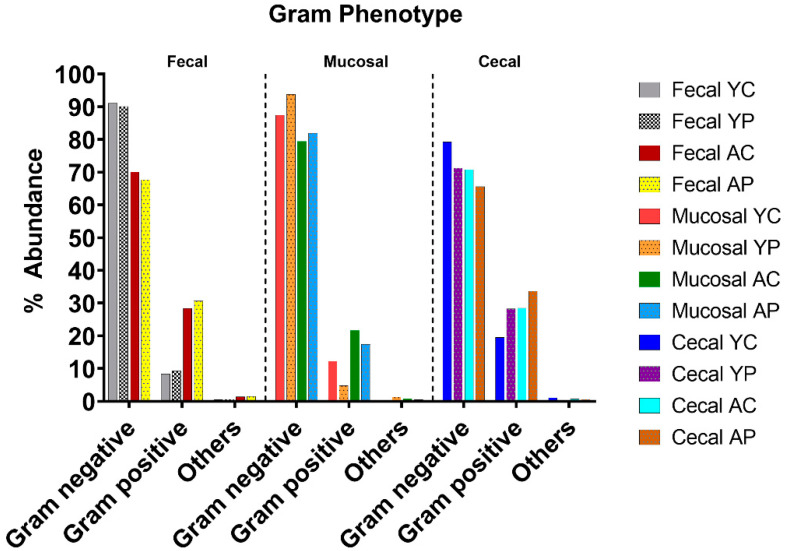
Gram-negative taxa differences between fecal, mucosal, and cecal samples in young and aging mice revealed by functional metagenomics analysis. (YC) Young control group, (YP) young probiotic group, (AC) aging control group, and (AP) aging probiotic group.

To understand the Gram phenotype in all 12 groups, we utilized taxonomic-to-phenotype functional analysis. Overall, the predominant groups in the microbiome were Gram-negative (Proteobacteria), compared to Gram-positive bacteria in the control groups (Figure 4). *L. acidophilus* DDS-1 supplementation had a marginal effect on the Gram-negative bacterial levels in the treatment groups, but noticeable changes were observed in cecal samples.

### 3.4. Age-Related Fecal Microbial Co-Occurrence Network Changes with L. acidophilus DDS-1

To understand the complex microbial structure among the study groups, we performed microbial co-occurrence analysis on fecal, mucosal, and cecal samples in young and aging mice with and without probiotic supplementation.

#### 3.4.1. Phylum-Level Fecal Co-Occurrences

**Figure 5:**
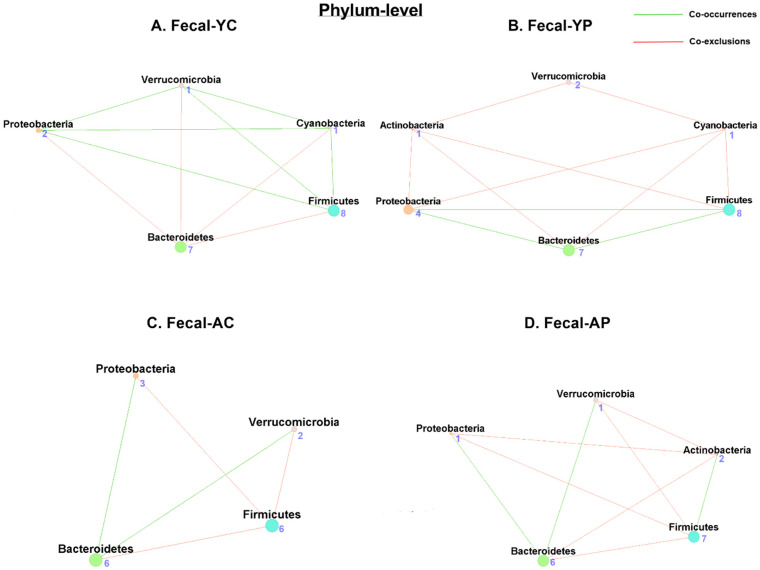
Microbial co-occurrence in the fecal microbiome in young and aging mice at the phylum level. Co-occurrence networks were constructed based on relative abundance profiles using Kendall’s Tau correlation analysis in young control (**A**), young probiotic (**B**), aging control (**C**), and aging probiotic (**D**) groups. Each node represents a phylum. Each edge indicates the sign of the association (green = positive (co-occurrences), red = negative (co-exclusions)). The thickness of the nodes represents the level of association between taxa.

Our network analysis on fecal samples revealed 5 nodes and 10 associations (6 co-occurrences and 4 co-exclusions) in F-YC (Figure 5A), while F-YP had 6 nodes and 11 associations (3 co-occurrences and 8 co-exclusions) (Figure 5B). Firmicutes, Verrucomicrobia, Proteobacteria, and Cyanobacteria shared the phyla with the most co-occurrences in F-YC, while Bacteroidetes had the most co-exclusions (Table S2). The phyla with the most co-occurrences in F-YP were Actinobacteria and Cyanobacteria, and Bacteroidetes has the most co-exclusion. In comparison, there were 4 nodes and 5 associations (2 co-occurrences and 3 co-exclusions) in F-AC (Figure 5C), and 5 nodes and 9 associations (3 co-occurrences and 6 co-exclusions) in F-AP (Figure 5D), while F-AC was less connected and phylum Bacteroidetes had the most co-occurrences, and Firmicutes had the most co-exclusions. Remarkably, F-AP was more connected than F-AC and phylum Bacteroidetes had the most co-occurrences, and Proteobacteria and Firmicutes had the most co-exclusions (Figure S2).

#### 3.4.2. Genus-Level Fecal Co-Occurrences

**Figure 6:**
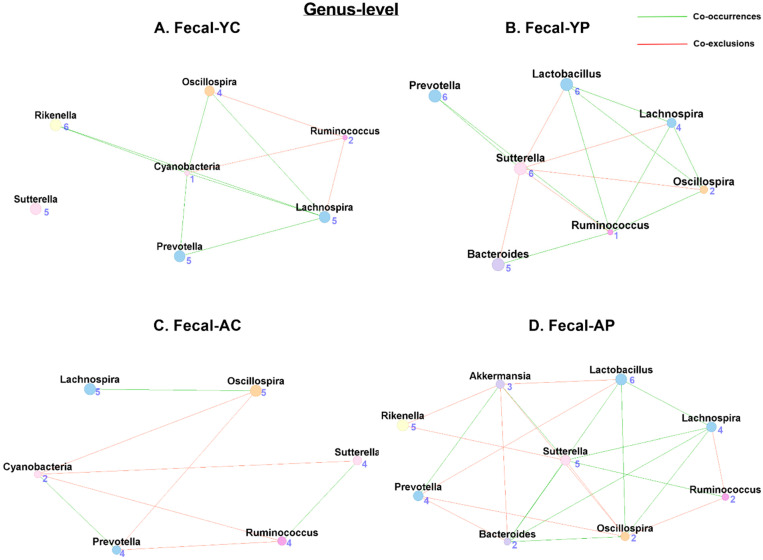
Microbial co-occurrence in the fecal microbiome in young and aging mice at the genus level. Co-occurrence networks were constructed based on relative abundance profiles using Kendall’s Tau correlation analysis in young control (**A**), young probiotic (**B**), aging control (**C**), and aging probiotic (**D**) groups. Each node represents a phylum. Each edge indicates the sign of the association (green = positive (co-occurrences), red = negative (co-exclusions)). The thickness of the nodes represents the level of association between taxa.

At the genus level, both F-YC (Figure 6A) and F-YP (Figure 6B) had 7 nodes, F-YC had 9 associations (6 co-occurrences and 3 co-exclusions), while F-YP had 13 associations (8 co-occurrences and 5 co-exclusions) (Table S2). In comparison, F-AC (Figure 6C) had 6 nodes and 8 associations only (3 co-occurrences and 5 co-exclusions), while F-AP (Figure 6D) had 9 nodes and 20 associations (10 co-occurrences and 10 co-exclusions). *Lactobacillus* taxon were absent in both of the control groups (Table 1). However, after 4 weeks of DDS-1 supplementation, the *Lactobacillus* node had 3 co-occurrences and 1 co-exclusion in F-YP, while F-AP had 3 co-occurrences and 2 co-exclusions associated with the *Lactobacillus* node (Figure S2).

### 3.5. Age-Related Mucosal Microbial Co-Occurrence Network Changes with L. acidophilus DDS-1

#### 3.5.1. Phylum-Level Mucosal Co-Occurrences

**Figure 7:**
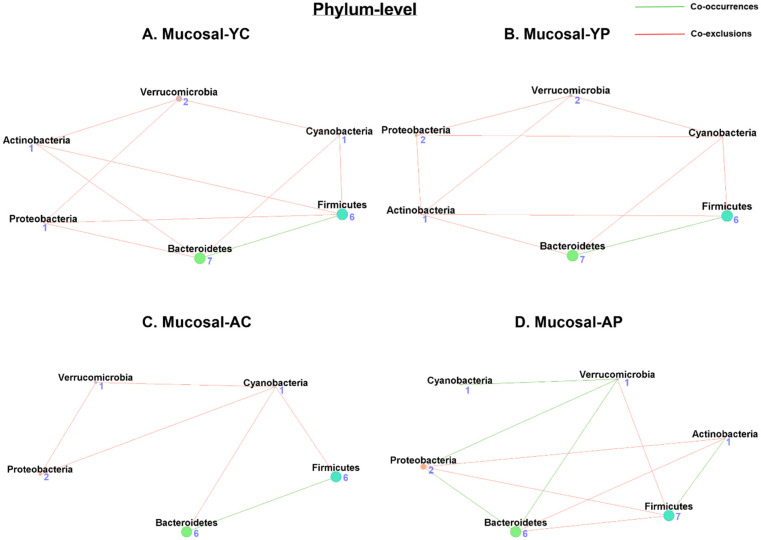
Microbial co-occurrence in the mucosal microbiome in young and aging mice at the phylum level. Co-occurrence networks were constructed based on relative abundance profiles using Kendall’s Tau correlation analysis in young control (**A**), young probiotic (**B**), aging control (**C**), and aging probiotic (**D**) groups. Each node represents a phylum. Each edge indicates the sign of the association (green = positive (co-occurrences), red = negative (co-exclusions)). The thickness of the nodes represents the level of association between taxa.

Our network analysis of the mucosal samples showed 6 nodes and 10 associations (1 co-occurrence and 9 co-exclusions) in M-YC (Figure 7A), while M-YP (Figure 7B) had 6 nodes and 10 associations (1 co-occurrence and 9 co-exclusions). The phyla with the most co-occurrences in M-YC was Bacteroidetes, while Firmicutes, Verrucomicrobia, Proteobacteria, and Cyanobacteria shared the most co-exclusions, a pattern similar to M-YP. We observed only 5 nodes and 6 associations (1 co-occurrence and 5 co-exclusions) in M-AC (Figure 7C), while M-AP (Figure 7D) had 6 nodes and 10 associations (5 co-occurrences and 5 co-exclusions). However, in M-AC, the Bacteroidetes phylum had a higher level of association as well as the most co-occurrences, and Cyanobacteria had the most co-exclusions. However, the M-AP structure improved, with the Verrucomicrobia phylum having the most co-occurrences, and the phylum Firmicutes had the most co-exclusions (Table S3).

#### 3.5.2. Genus-Level Mucosal Co-Occurrences

**Figure 8:**
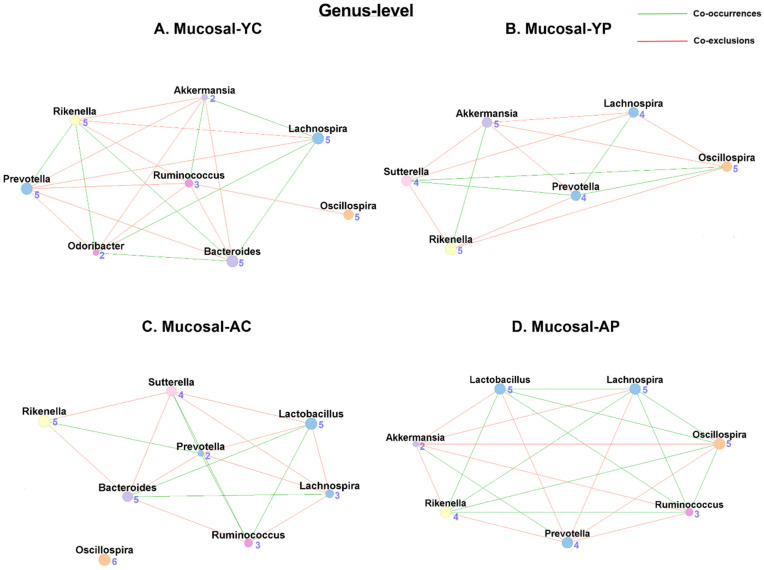
Microbial co-occurrence in the mucosal microbiome in young and aging mice at the genus level. Co-occurrence networks were constructed on the basis of the relative abundance profiles of fecal microbes using Kendall’s Tau correlation analysis in young control (**A**), young probiotic (**B**), aging control (**C**), and aging probiotic (**D**) groups. Each node represents a phylum. Each edge indicates the sign of the association (green = positive (co-occurrences), red = negative (co-exclusions)). The thickness of the nodes represents the level of association between taxa.

Mucosal genus-level co-occurrence has shown 8 nodes and 17 associations (7 co-occurrences and 10 co-exclusions) in M-YC (Figure 8A), and 6 nodes and 13 associations (5 co-occurrences and 9 co-exclusions) in M-YP (Figure 8B). In the M-AC (Figure 8C), we observed 8 nodes and 17 associations (6 co-occurrences and 11 co-exclusions), while the M-AP (Figure 8D) had 7 nodes and 18 associations (9 co-occurrences and 9 co-exclusions). Unlike fecal samples, we also found *Lactobacillus* taxon in M-AC (2 co-occurrences and 3 co-exclusions) but not in M-YC. Post-DDS-1 supplementation, the *Lactobacillus* taxon was only found in M-AP (4 co-occurrences and 2 co-exclusions) (Table 1 and Table S3).

### 3.6. Age-Related Cecal Microbial Co-Occurrence Network Changes with DDS-1

#### 3.6.1. Phylum-Level Cecal Co-Occurrences

**Figure 9:**
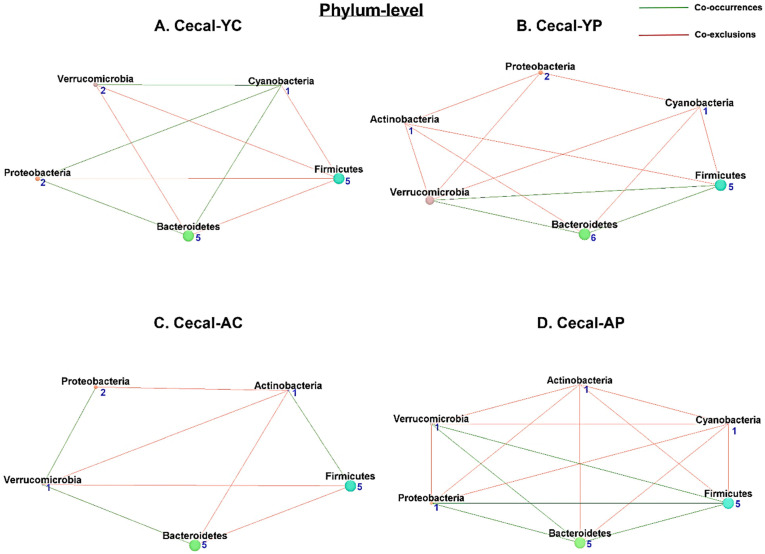
Microbial co-occurrence in the cecal microbiome in young and aging mice at the phylum level. Co-occurrence networks were constructed based on relative abundance profiles of fecal microbes using Kendall’s Tau correlation analysis in young control (**A**), young probiotic (**B**), aging control (**C**), and aging probiotic (**D**) groups. Each node represents a phylum. Each edge indicates the sign of the association (green = positive (co-occurrences), red = negative (co-exclusions)). The thickness of the nodes represents the level of association between taxa.

Cecal microbial network analysis has shown 5 nodes and 9 associations (4 co-occurrences and 5 co-exclusions) in the C-YC group (Figure 9A), and the C-YP group (Figure 9B) had 6 nodes and 12 associations (3 co-occurrences and 9 co-exclusions). The phylum with the most co-occurrences was Cyanobacteria, and the Firmicutes phylum had the most co-exclusions in the C-YC group, while Actinobacteria and Cyanobacteria had the most co-occurrences in C-YP. We observed 5 nodes and 8 associations (3 co-occurrences and 5 co-exclusions) in the C-AC group (Figure 9C); in comparison, C-AP (Figure 9D) had 6 nodes and 15 associations (5 co-occurrences and 10 co-exclusions). In C-AC, the Verrucomicrobia phylum had the most co-occurrences, and the Actinobacteria phylum had the most co-exclusions. Firmicutes and Bacteroidetes had the most co-occurrences in the C-AP group. Surprisingly, the Actinobacteria phylum, together with the Cyanobacteria phylum, had the most co-exclusions in both the C-YP and C-AP groups (Table S4).

#### 3.6.2. Genus-Level Cecal Co-Occurrences

**Figure 10:**
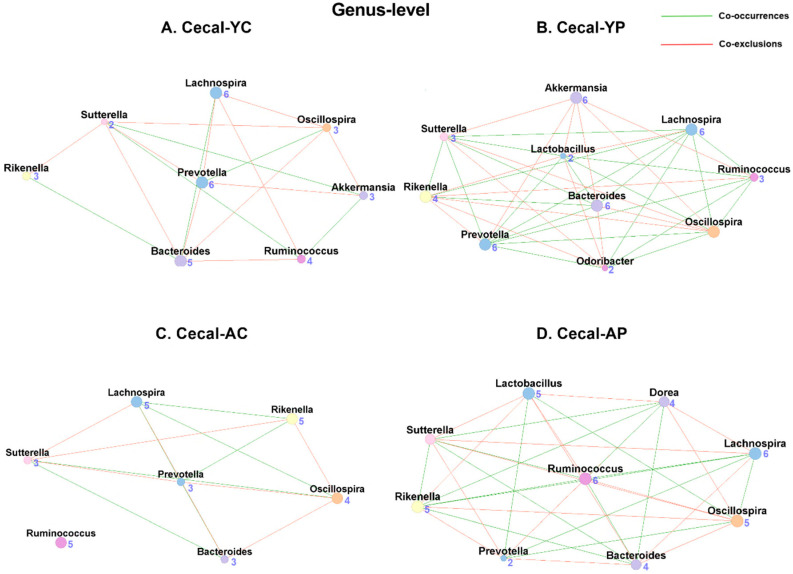
Microbial co-occurrence in the cecal microbiome in young and aging mice at the genus level. Co-occurrence networks were constructed based on relative abundance profiles of fecal microbes using Kendall’s Tau correlation analysis in young control (**A**), young probiotic (**B**), aging control (**C**), and aging probiotic (**D**) groups. Each node represents a genus. Each edge indicates the sign of the association (green = positive (co-occurrences), red = negative (co-exclusions)). The thickness of the nodes represents the level of association between taxa.

In cecal samples, we found 8 nodes and 21 associations (6 co-occurrences and 11 co-exclusions) in the C-YC group (Figure 10A), while the C-YP group (Figure 10B) had 10 nodes and 33 associations (18 co-occurrences and 15 co-exclusions). In aging groups, C-AC (Figure 10C) had only 7 nodes and 14 associations (5 co-occurrences and 9 co-exclusions), while C-AP (Figure 10D) had 9 nodes and 29 associations (14 co-occurrences and 15 co-exclusions), suggesting DDS-1-induced modulations. Similar to fecal samples, the *Lactobacillus* taxon was absent in the control groups (Table 1). With DDS-1 supplementation, the *Lactobacillus* taxon was found in both of the probiotic groups, C-YP (5 co-occurrences and 4 co-exclusions) and C-AP (2 co-occurrences and 5 co-exclusions) (Table S4).

## 4. Discussions

Here, we focused on the microbial co-occurrence analysis of the gut microbiota, to investigate aging-related changes of the microbiome structure with and without *L. acidophilus* DDS-1 supplementation. To the best of our knowledge, this is the first study to show differences in microbial co-occurrences in fecal, mucosal, and cecal samples in young and aging mice with or without probiotic supplementation. Our work provides novel insights on probiotic-induced modulations of gut microbial co-occurrence networks and the overall microbiome community structure. We found distinct microbial networks with age and across the sample types. Moreover, aging controls had generally higher abundances of Proteobacteria (Figure S1A). *L. acidophilus* DDS-1 supplementation helped modulate the biological networks at phylum and genus levels. Particularly, DDS-1 significantly increased the abundance of both the genera *Lactobacillus* and *Akkermansia*, and reduced Proteobacteria networks, thereby increasing the co-occurrence networks and maintaining well-connected networks (compared to controls) with age (Figure S1B). This work demonstrates the importance of location-specific (fecal, mucosal, and cecal) microbiome comparisons in understanding age-related dysbiosis as well as potential probiotic modulation. Additionally, our work supports the role of next-generation analysis, such as ‘microbial co-occurrences’, in framing the ecological role and dynamics of the microbial community.

In line with previous studies on the aging microbiome, we have found differences between control and probiotic groups [25,26]. The microbiome composition was found to be different throughout the sample types with age. Bacteroidetes, Firmicutes, *Verrucomicrobia*, and Proteobacteria were the most dominant phyla. We observed more Gram-negative bacteria compared to Gram-positive in young control mice. Further, noticeable reductions in Gram-negative bacteria were observed only in the cecal probiotic groups (C-YP and C-AP). Gram-negative bacteria, such as Proteobacteria, were characterized by lipopolysaccharide (endotoxin) on the outer cell membrane. Increased Proteobacteria in older people, mice, as well as NHPs has led to higher microbial translocation and upregulation of plasma pro-inflammatory immune markers [14,18,27,28,29]. Further, Verrucomicrobia phylum levels were differentially abundant across the control groups. Most of the groups, except C-AC, had noticeable levels of the Verrucomicrobia phylum, which includes major beneficial genera such as *Akkermansia*. At the genus level, S24-7, *Akkermansia,* and order Clostridiales (*Ruminococcus*) were dominant genera. *L. acidophilus* DDS-1 supplementation helped to moderately reduce Proteobacteria and further improved *Akkermansia* (YP vs. AP) and *Lactobacillus* levels. Here, the small differences with DDS-1 treatment on Gram-negative bacteria were smaller than the effect of age itself, as observed in the Gram phenotype analysis. We have previously reported on modulation of the microbiome, the metabolome by increasing SCFA production, and reducing inflammation in mice [24]. The current study demonstrated that the beta diversity was significantly distinct, and dissimilarities existed between the 12 groups. Beta diversity clustering by richness and abundance corresponding to probiotic supplementation is indicative of a strong taxonomic effect, which influenced the gut microbial structure. These differences may be of interest as it relates to controlling dysbiosis in aging populations [4]. Though we did not find any differences in microbiome in male and female mice in this study, previous research has noted that there are sex differences [30,31]. Hence, further research in larger samples is recommended.

Beyond taxonomic and diversity profiles, there are gaps in knowledge about the composition and ecological structure of microbial communities [32]. Our recent study of NHPs provided evidence regarding the age-related mucosal microbiome structure via novel microbial co-occurrence analysis [16]. Microbiome structure was shown to be defined by their co-occurrence (positive) and co-exclusion (negative) networks, and these networks were less connected in older NHPs. Further, we found that overall, microbiome community dynamics were driven by the presence or absence of taxa, suggesting a niche process of dysbiosis with age. Similarly, we have previously reported dysbiosis in the fecal, mucosal, and cecal microbiomes in aging mice compared to young mice [25,26]. After four weeks of dietary supplementation with *L. acidophilus* DDS-1, we observed microbiome modulation in young and aging mice. We found distinct interactive biological networks in all 12 groups. Particularly at the phylum level, we observed evidence of age-related dysbiosis impacts on aging controls. The networks from each sample type had a differing number of associations, with YC having numerically more associations and being well-connected compared to AC. Moreover, some of the associations appeared to be paired (YC vs. AC) with similar nodes, suggesting that aging significantly modulated the network structure and composition. Firmicutes and Bacteroidetes, being the most dominant and widely studied phyla, had fewer associations in AC compared to YC. Conversely, less dominant phyla such as Cyanobacteria and Verrucomicrobia had numerically higher associations across both age groups. As mentioned above, most of the AC groups had more association networks of Proteobacteria, which appeared to be important taxa altering the co-occurrences. In contrast, all the probiotic groups were more connected regardless of age, and had a reduction in Proteobacteria abundance, notably more in cecal samples. Taken together, DDS-1 supplementation demonstrated a potential to reduce the relative abundance and alter microbial interactions of opportunistic pathogens such as Proteobacteria.

To enhance our understanding, we performed a similar analysis at the genus level to further investigate probiotic modulation of co-occurrences networks. In most of the control groups regardless of age or sample type, the *Lactobacillus* genus was absent, except for M-AC. The M-AC group had two Lactobacillus-related co-occurrences and three co-exclusions; these could be location-specific findings, and further research is needed in colonic mucosal samples in aging mice to confirm these findings. Post-DDS-1 supplementation, we found increases in *Lactobacillus* abundance along with increases in *Akkermansia* (more in YP groups), though only in F-AP. We observed the associations of *Lactobacillus* taxa increase in the DDS-1-treated groups, except M-YP. Of all the *Lactobacillus* associations, the *Lactobacillus–Oscillospira* co-occurrence association was found to be the most common between the groups. Interestingly, *Oscillospira* spp. was shown to be beneficial in metabolic conditions and may have anti-inflammatory properties [33,34]. Further, potential pathobionts such as *Ruminococcus* had more co-exclusions, and influence the co-occurrence network of the aging controls. *Ruminococcus* members such as *R. gnavus* species were found to stimulate pro-inflammatory responses in Crohn’s disease [35]. However, *L. acidophilus* DDS-1 supplementation was shown to balance the *Ruminococcus* associations with more co-occurrences. Although the S24-7 genus was the most dominant in taxonomical profiles, its role in defining networks was absent. Conversely, less dominant genera such as *Bacteroides*, *Lachnospira,* and *Oscillospira,* as well as Verrucomicrobia, had higher associations across the groups, suggesting the importance of the absence or presence of particular genera in modulating microbial community, but not based on the taxonomic structure [15,16,19,27,32,34].

One of the strengths of this study is the use of the novel co-occurrence analysis. These co-occurrence networks are independent of taxonomic classifications and do not depend on the major microbial phyla or genera. All the of association networks were produced after FDR correction (*q* < 0.005) was performed to eliminate spurious findings. This supports the notion that microbiome community structure is determined by niche-specific factors (nutrients, oxygen levels, and pH) and driven by functional characteristics, but not phylogeny [15]. Overall, the approach enabled us to conclude that the gut microbes are inclined to co-occur or co-exclude more than is expected by chance. Next, by utilizing the fecal, cecal, and mucosal samples, we were able to investigate the site-specific microbiome differences between young and aging mice. Further, the use of the *L. acidophilus* DDS-1 intervention enabled us to understand the ability of probiotics to modulate the intestinal microbiome structure, particularly in aging mice as compared to younger mice. This study, along with our previous works, provided information on microbial dysbiotic changes with aging, as well as the development of dysbiotic signatures or drift that may begin at middle age [2,4,36,37,38,39,40]. Additionally, our work suggests that the use of *L. acidophilus* DDS-1 at this mid-life stage may be beneficial to maintain healthy microbiomes in later life. The aging animals in our study correspond to middle age, but not necessarily centenarian, such as oldest old or aged mice, which may explain the microbiome changes observed in this study. Our co-occurrence network analysis has provided important information on the complexity of microbial structures and their interactions; however, the work is limited to the genus level. Future studies should be performed using high-resolution metagenomics to identify species/strain-level changes. Incorporating probiotics such as *L. acidophilus* DDS-1 in the diet can modulate the microbiome in mice. The use of a more translationally relevant NHP model to assess the networks with probiotic supplementation will provide more insights.

## 5. Conclusions

The present study evaluated the probiotic *L. acidophilus* DDS-1 on its ability to modulate microbial co-occurrences in fecal, mucosal, and cecal samples of young and aging mice. DDS-1 helped modulate age-related microbial co-occurrence networks in specific ways relative to intestinal location. In addition to taxonomical changes, *L. acidophilus* DDS-1 supplementation influenced fecal and mucosal microbial co-occurrences with edges that are more positive and increased networks. The microbial co-occurrence network approach has provided us with novel insights into the potential functional role of DDS-1 on key microbial taxa in microbial communities. Future studies should utilize microbial co-occurrence analysis as a next-generation tool to enhance our understandings of probiotic-induced structural changes in the overall microbiome community during various age-related health conditions.

## Figures and Tables

**Table 1:** Summary of number of associations based on the *Lactobacillus* genus in all four groups across the sample types.

Group	Co-Occurrences	Associations	Co-Exclusions	Associations
F-YC	0		0	
F-YP	3	*Lactobacillus-Lachnospira* *Lactobacillus-Oscillospira* *Lactobacillus-Ruminococcus*	1	*Lactobacillus-Sutterella*
F-AC	0		0	
F-AP	3	*Lactobacillus-Lachnospira* *Lactobacillus-Oscillospira* *Lactobacillus-Sutterella*	2	*Lactobacillus-Prevotella* *Lactobacillus-Akkermansia*
M-YC	0		0	
M-YP	0		0	
M-AC	2	*Lactobacillus-Ruminoccocus* *Lactobacillus-Bacteroides*	3	*Lactobacillus-Sutterella* *Lactobacillus-Lachnospira* *Lactobacillus-Prevotella*
M-AP	4	*Lactobacillus-Lachnospira* *Lactobacillus-Oscillospira* *Lactobacillus-Ruminococcus* *Lactobacillus-Rikenella*	2	*Lactobacillus-Prevotella* *Lactobacillus-Akkermansia*
C-YC	0		0	
C-YP	5	*Lactobacillus-Prevotella* *Lactobacillus-Bacteroides* *Lactobacillus-Ruminococcus* *Lactobacillus-Oscillospira* *Lactobacillus-Sutterella*	4	*Lactobacillus-Akkermansia* *Lactobacillus-Odoribacter* *Lactobacillus-Lachnospira* *Lactobacillus-Rikenella*
C-AC	0		0	
C-AP	2	*Lactobacillus-Oscillospira* *Lactobacillus-Prevotella*	5	*Lactobacillus-Sutterella* *Lactobacillus-Dorea* *Lactobacillus-Bacteroides* *Lactobacillus-Ruminococcus* *Lactobacillus-Rikenella*

YC = young control, YP = young probiotic, AC = aging control, AP = aging probiotic, F = fecal, M = mucosal, and C = cecal.

## Data Availability

Sequence data have been deposited publicly in the Figshare database (DOI: https://10.6084/m9.figshare.17019947 accessed on 8 February 2022).

## Supplementary Materials

The following are available online at https://www.mdpi.com/article/10.3390/nu14050977/s1, Figure S1: Bacterial taxa identified to be differentially abundant by linear discrimination analysis (LDA) effect size (LEfSe, log LDA > 2.0) analysis in all 4 samples at the phylum level (A) and genus level (B). The values are shown as the mean ± SEM. * Significant differences with *p* < 0.05. Figure S2: Summary of co-occurrence and co-exclusion analysis between young and aging mice at the phylum and genus levels in fecal (A,B), mucosal (C,D), and cecal (E,F) samples. (YC) Young control group, (YP) young probiotic group, (AC) aging control group, and (AP) aging probiotic group. Table S1: Nutritional composition details of Barastoc mice standard chow. Table S2: Summary of fecal microbiome co-occurrences between young and aging mice at phylum and genus levels based on Kendall’s Tau correlation analysis, after which FDR (*q* < 0.05) correction was performed. Table S3: Summary of mucosal microbiome co-occurrences between young and aging mice at phylum and genus levels based on Kendall’s Tau correlation analysis, after which FDR (*q* < 0.05) correction was performed. Table S4: Summary of cecal microbiome co-occurrences between young and aging mice at phylum and genus levels based on Kendall’s Tau correlation analysis, after which FDR (*q* < 0.05) correction was performed.
