# Supplementary material for: Lactobacillus acidophilus DDS-1 Modulates the Gut Microbial Co-Occurrence Networks in Aging Mice
Source: Nutrients. 2022 Feb 25;14(5):977. doi: 10.3390/nu14050977 (PMC8912519; doi:10.3390/nu14050977)

Lactobacillus acidophilus DDS-1 Modulates Gut Microbial Co-Occurrence Networks in Aging Mice

Ravichandra Vemuri[1, 3] Christopher J. Martoni[2], Kylie Kavanagh[1,4] and Rajaraman Eri[3]

[1]Department of Pathology, Section on Comparative Medicine, Wake Forest School of Medicine, Medical Center Boulevard, Winston-Salem, NC 27157, USA

[2]UAS Laboratories, Madison, Wisconsin, WI, 54401 USA

[3]College of Health and Medicine, School of Health Sciences, University of Tasmania, Launceston, 7248, TAS, Australia

[4]Department of Biomedicine, University of Tasmania, Hobart, 7000, TAS, Australia

Correspondence:

Ravichandra Vemuri and Rajaraman Eri

Department of Pathology, Section of Comparative Medicine, Wake Forest School of Medicine, Medical Center Boulevard, Winston-Salem, NC 27157, USA.

College of Health and Medicine, School of Health Sciences, University of Tasmania, Launceston, 7248, TAS, Australia

Table S1: Nutritional composition details of Barastoc mice standard chow

Contents	
Minimal crude protein 20 %	Vitamin A 15 IU/g
Minimal crude fat 6 %	Vitamin D3 2 IU/g
Crude fibre 3.2 %	Vitamin E 260 mg/kg
Acid detergent fibre 4.4 %	Vitamin K3 55 mg/kg
Neutral detergent fibre 10.4 %	Vitamin B1 64 mg/kg
Digestible energy 12.8 MJ/kg	Vitamin B2 48 mg/kg
Calcium 1.14 %	Vitamin B6 30 mg/kg
Phosphorus 0.94 %	Vitamin B12 0.08 mg/kg
Sodium 0.35 %	Niacin 400 mg/kg
Potassium 0.82 %	Panto 220 mg/kg
Chloride 0.58 %	Biotin 1.48 mg/kg
Magnesium 0.24 %	Folic 11 mg/kg
Lysine 1.11 %	Iron 51 mg/kg
Methionine 0.37 %	Zinc 60 mg/kg
Linoleic 1.52 %	Manganese 120 mg/kg
Starch 29 %	Copper 10 mg/kg
Vitamin A 15 IU/g	Selenium 0.1 mg/kg
Vitamin D3 2 IU/g	Molybdenum 0.4 mg/kg
Vitamin E 260 mg/kg	Cobalt 0.6 mg/kg
Vitamin K3 55 mg/kg	Iodine 1 .4 mg/kg

Table S2: Summary of fecal microbiome co-occurrences between young and aging mice at phylum and genus levels based on Kendall's Tau correlation analysis after which FDR (q<0.05) correction was performed.

Young Control (Phylum)				Young Probiotic (Phylum)			
**Taxon1**	**Taxon2**	**Correlation (p<0.05)**	**Association**	**Taxon1**	**Taxon2**	**Correlation (p<0.05)**	**Association**
Firmicutes	Cyanobacteria	1	Positive	Firmicutes	Bacteroidetes	1	Positive
Firmicutes	Verrucomicrobia	0.6	Positive	Firmicutes	Proteobacteria	0.6	Positive
Firmicutes	Proteobacteria	1	Positive	Firmicutes	Cyanobacteria	-0.6	Negative
Firmicutes	Bacteroidetes	-1	Negative	Firmicutes	Actinobacteria	-0.5	Negative
Bacteroidetes	Cyanobacteria	-1	Negative	Bacteroidetes	Proteobacteria	0.6	Positive
Bacteroidetes	Firmicutes	-1	Negative	Bacteroidetes	Cyanobacteria	0.5	Negative
Bacteroidetes	Verrucomicrobia	-0.6	Negative	Bacteroidetes	Actinobacteria	0.3	Negative
Bacteroidetes	Proteobacteria	-1	Negative	Bacteroidetes	Firmicutes	1	Positive
Verrucomicrobia	Cyanobacteria	0.5	Positive	Verrucomicrobia	Cyanobacteria	-0.3	Negative
Verrucomicrobia	Firmicutes	0.6	Positive	Verrucomicrobia	Actinobacteria	-1	Negative
Verrucomicrobia	Proteobacteria	0.6	Positive	Proteobacteria	Firmicutes	1	Positive
Verrucomicrobia	Bacteroidetes	-1	Negative	Proteobacteria	Bacteroidetes	1	Positive
Proteobacteria	Cyanobacteria	0.6	Positive	Proteobacteria	Cyanobacteria	-1	Negative
Proteobacteria	Firmicutes	1	Positive	Proteobacteria	Actinobacteria	-0.5	Negative
Proteobacteria	Verrucomicrobia	0.5	Positive	Cyanobacteria	Firmicutes	-0.6	Negative
Proteobacteria	Bacteroidetes	-1	Negative	Cyanobacteria	Proteobacteria	-0.5	Negative
Cyanobacteria	Firmicutes	1	Positive	Cyanobacteria	Bacteroidetes	-1	Negative
Cyanobacteria	Proteobacteria	0.5	Positive	Cyanobacteria	Actinobacteria	-0.7	Negative
Cyanobacteria	Bacteroidetes	-1	Negative	Actinobacteria	Firmicutes	-0.5	Negative

Cyanobacteria	Verrucomicrobia	0.5	Positive	Actinobacteria	Proteobacteria	-0.5	Negative
				Actinobacteria	Bacteroidetes	-1	Negative
				Actinobacteria	Cyanobacteria	-0.5	Negative
**Young Control (Genus)**				**Young Probiotic (Genus)**			
**Taxon1**	**Taxon2**	**Correlation (p<0.05)**	**Association**	**Taxon1**	**Taxon2**	**Correlation (p<0.05)**	**Association**
Prevotella	Cyanobacteria	1	Positive	Prevotella	Sutterella	1	Positive
Prevotella	Lachnospira	0.6	Positive	Prevotella	Ruminococcus	0.6	Positive
Lachnospira	Prevotella	0.6	Positive	Lachnospira	Lactobacillus	1	Positive
Lachnospira	Cyanobacteria	0.3	Positive	Lachnospira	Ruminococcus	0.6	Positive
Lachnospira	Ruminococcus	-0.5	Negative	Lachnospira	Sutterella	-1	Negative
Lachnospira	Rikenella	1	Positive	Lachnospira	Oscillospira	0.5	Positive
Lachnospira	Oscillospira	0.5	Positive	Lactobacillus	Lachnospira	1	Positive
Ruminococcus	Lachnospira	-1	Negative	Lactobacillus	Ruminococcus	0.6	Positive
Ruminococcus	Oscillospira	-0.5	Negative	Lactobacillus	Sutterella	-1	Negative
Ruminococcus	Cyanobacteria	-0.3	Negative	Lactobacillus	Oscillospira	0.3	Positive
Cyanobacteria	Oscillospira	0.6	Positive	Ruminococcus	Prevotella	0.6	Positive
Cyanobacteria	Rikenella	0.5	Positive	Ruminococcus	Lachnospira	0.6	Positive
Cyanobacteria	Ruminococcus	-0.3	Negative	Ruminococcus	Lactobacillus	0.6	Positive
Cyanobacteria	Lachnospira	0.3	Positive	Ruminococcus	Sutterella	-0.6	Negative
Cyanobacteria	Oscillospira	1	Positive	Ruminococcus	Bacteroides	0.3	Positive
Cyanobacteria	Prevotella	1	Positive	Sutterella	Ruminococcus	-0.6	Negative
Rikenella	Cyanobacteria	0.5	Positive	Sutterella	Lactobacillus	-1	Negative
Rikenella	Lachnospira	1	Positive	Sutterella	Prevotella	1	Positive
Oscillospira	Lachnospira	0.5	Positive	Sutterella	Lachnospira	0.5	Positive
Oscillospira	Cyanobacteria	0.6	Positive	Sutterella	Bacteroides	-1	Negative
Oscillospira	Ruminococcus	-0.5	Negative	Sutterella	Oscillospira	-0.5	Negative
				Bacteroides	0.3	Ruminococcus	Positive
				Bacteroides	-1	Sutterella	Negative
				Oscillospira	Sutterella	-0.5	Negative

				Oscillospira	Lactobacillus	0.3	Positive
				Oscillospira	Lachnospira	0.5	Positive
				Oscillospira	Ruminococcus	0.3	Positive
**Aging Control (Phylum)**				**Aging Probiotic (Phylum)**			
**Taxon1**	**Taxon2**	**Correlation ($p<0.05$)**	**Association**	**Taxon1**	**Taxon2**	**Correlation ($p<0.05$)**	**Association**
Firmicutes	Proteobacteria	-0.5	Negative	Firmicutes	Actinobacteria	0.3	Positive
Firmicutes	Bacteroidetes	-1	Negative	Firmicutes	Proteobacteria	-0.3	Negative
Firmicutes	Verrucomicrobia	-0.3	Negative	Firmicutes	Bacteroidetes	-1	Negative
Bacteroidetes	Firmicutes	-1	Negative	Firmicutes	Verrucomicrobia	-0.5	Negative
Bacteroidetes	Verrucomicrobia	0.5	Positive	Bacteroidetes	Firmicutes	-1	Negative
Bacteroidetes	Proteobacteria	0.3	Positive	Bacteroidetes	Verrucomicrobia	0.3	Positive
Verrucomicrobia	Firmicutes	-0.3	Negative	Bacteroidetes	Proteobacteria	0.5	Positive
Verrucomicrobia	Bacteroidetes	0.5	Positive	Bacteroidetes	Actinobacteria	-0.3	Negative
Proteobacteria	Bacteroidetes	0.3	Positive	Proteobacteria	Firmicutes	-0.3	Negative
Proteobacteria	Firmicutes	-0.5	Negative	Proteobacteria	Bacteroidetes	0.5	Positive
				Proteobacteria	Actinobacteria	-0.3	Negative
				Verrucomicrobia	Bacteroidetes	0.3	Positive
				Verrucomicrobia	Firmicutes	-0.5	Negative
				Verrucomicrobia	Actinobacteria	-0.3	Negative
				Actinobacteria	Verrucomicrobia	-0.3	Negative
				Actinobacteria	Bacteroidetes	-0.3	Negative
				Actinobacteria	Firmicutes	0.3	Positive
				Actinobacteria	Proteobacteria	-0.3	Negative
**Aging Control (Genus)**				**Aging Probiotic (Genus)**			

**Taxon1**	**Taxon2**	**Correlation (p<0.05)**	**Association**	**Taxon1**	**Taxon2**	**Correlation (p<0.05)**	**Association**
Prevotella	Cyanobacteria	0.5	Positive	Prevotella	Akkermansia	0.6	Positive
Prevotella	Ruminococcus	-0.3	Negative	Prevotella	Bacteroides	-0.3	Negative
Prevotella	Oscillospira	-1	Negative	Prevotella	Lactobacillus	-1	Negative
Cyanobacteria	Prevotella	0.5	Positive	Prevotella	Oscillospira	-0.5	Negative
Cyanobacteria	Ruminococcus	-0.5	Negative	Akkermansia	Prevotella	0.6	Positive
Cyanobacteria	Oscillospira	-1	Negative	Akkermansia	Bacteroides	-0.5	Negative
Cyanobacteria	Sutterella	-0.5	Negative	Akkermansia	Lactobacillus	-1	Negative
Ruminococcus	Prevotella	-0.3	Negative	Akkermansia	Oscillospira	-0.6	Negative
Ruminococcus	Cyanobacteria	-0.5	Negative	Akkermansia	Sutterella	1	Positive
Ruminococcus	Sutterella	1	Positive	Lactobacillus	Prevotella	-1	Negative
Oscillospira	Prevotella	-1	Negative	Lactobacillus	Akkermansia	-1	Negative
Oscillospira	Cyanobacteria	-1	Negative	Lactobacillus	Sutterella	1	Positive
Oscillospira	Lachnospira	1	Positive	Lactobacillus	Oscillospira	0.6	Positive
Lachnospira	Oscillospira	1	Positive	Lactobacillus	Lachnospira	1	Positive
				Sutterella	Lactobacillus	1	Positive
				Sutterella	Akkermansia	1	Positive
				Sutterella	Bacteroides	0.6	Positive
				Sutterella	Oscillospira	-1	Negative
				Sutterella	Ruminococcus	0.5	Positive
				Sutterella	Lactobacillus	1	Positive
				Lachnospira	Lactobacillus	1	Positive
				Lachnospira	Bacteroides	0.6	Positive
				Lachnospira	Sutterella	1	Positive
				Lachnospira	Ruminococcus	-0.5	Negative
				Lachnospira	Oscillospira	1	Positive
				Oscillospira	Lachnospira	1	Positive
				Oscillospira	Sutterella	-1	Negative
				Oscillospira	Lactobacillus	0.6	Positive
				Oscillospira	Bacteroides	-0.4	Negative

				Bacteroides	Oscillospira	-0.4	Negative
				Bacteroides	Lachnospira	0.6	Positive
				Bacteroides	Sutterella	0.6	Positive
				Bacteroides	Ruminococcus	0.5	Positive
				Bacteroides	Akkermansia	-0.5	Negative
				Bacteroides	Prevotella	-0.3	Negative
				Rikenella	Akkermansia	-0.6	Negative
				Rikenella	Sutterella	-1	Negative
				Ruminococcus	Lachnospira	-0.5	Negative
				Ruminococcus	Sutterella	0.5	Positive
				Ruminococcus	Bacteroides	0.5	Positive

Table S3: Summary of mucosal microbiome co-occurrences between young and aging mice at phylum and genus levels based on Kendall's Tau correlation analysis after which FDR (q<0.05) correction was performed.

Young Control (Phylum)				Young Probiotic (Phylum)			
**Taxon1**	**Taxon2**	**Correlation (p<0.05)**	**Association**	**Taxon1**	**Taxon2**	**Correlation (p<0.05)**	**Association**
Firmicutes	Bacteroidetes	1	Positive	Firmicutes	Bacteroidetes	1	Positive
Firmicutes	Proteobacteria	-0.2	Negative	Firmicutes	Cyanobacteria	-0.5	Negative
Firmicutes	Cyanobacteria	-0.2	Negative	Firmicutes	Actinobacteria	-0.3	Negative
Firmicutes	Actinobacteria	-0.3	Negative	Bacteroidetes	Firmicutes	1	Positive
Bacteroidetes	Firmicutes	1	Positive	Bacteroidetes	Cyanobacteria	-0.5	Negative
Bacteroidetes	Proteobacteria	-0.3	Negative	Bacteroidetes	Actinobacteria	-0.5	Negative
Bacteroidetes	Cyanobacteria	-0.2	Negative	Actinobacteria	Bacteroidetes	-0.5	Negative
Bacteroidetes	Actinobacteria	-0.2	Negative	Actinobacteria	Firmicutes	-0.3	Negative
Proteobacteria	Firmicutes	-1	Negative	Actinobacteria	Proteobacteria	-0.5	Negative
Proteobacteria	Bacteroidetes	-1	Negative	Actinobacteria	Verrucomicrobia	-0.3	Negative
Proteobacteria	Verrucomicrobia	-0.5	Negative	Cyanobacteria	Firmicutes	-0.5	Negative
Verrucomicrobia	Proteobacteria	-0.5	Negative	Cyanobacteria	Bacteroidetes	-0.5	Negative
Verrucomicrobia	Cyanobacteria	-0.3	Negative	Cyanobacteria	Verrucomicrobia	-0.3	Negative
Verrucomicrobia	Actinobacteria	-0.3	Negative	Cyanobacteria	Proteobacteria	-0.3	Negative
Cyanobacteria	Firmicutes	-0.2	Negative	Proteobacteria	Cyanobacteria	-0.3	Negative
Cyanobacteria	Verrucomicrobia	-0.3	Negative	Proteobacteria	Actinobacteria	-0.5	Negative
Cyanobacteria	Bacteroidetes	-0.2	Negative	Proteobacteria	Verrucomicrobia	-0.5	Negative
Actinobacteria	Verrucomicrobia	-0.3	Negative	Verrucomicrobia	Proteobacteria	-0.5	Negative
Actinobacteria	Bacteroidetes	-0.2	Negative	Verrucomicrobia	Cyanobacteria	-0.3	Negative
Actinobacteria	Firmicutes	-0.3	Negative	Verrucomicrobia	Actinobacteria	-0.3	Negative

Young Control (Genus)				Young Probiotic (Genus)			
**Taxon1**	**Taxon2**	**Correlation ($p<0.05$)**	**Associatio n**	**Taxon1**	**Taxon2**	**Correlation ($p<0.05$)**	**Association**
Prevotella	Rikenella	0.6	Positive	Prevotella	Rikenella	-1	Negative
Prevotella	Odoribacter	-0.3	Negative	Prevotella	Oscillospira	0.6	Positive
Prevotella	Ruminococcus	-0.5	Negative	Prevotella	Ruminococcus	-0.5	Negative
Prevotella	Akkermansia	-0.3	Negative	Prevotella	Akkermansia	-1	Negative
Prevotella	Lachnospira	-1	Negative	Prevotella	Lachnospira	1	Positive
Prevotella	Bacteroides	-1	Negative	Prevotella	Sutterella	1	Positive
Ruminococcus	Prevotella	-0.5	Negative	Sutterella	Prevotella	1	Positive
Ruminococcus	Akkermansia	0.5	Positive	Sutterella	Akkermansia	-1	Negative
Ruminococcus	Odoribacter	-0.5	Negative	Sutterella	Lachnospira	-0.6	Negative
Ruminococcus	Bacteroides	-1	Negative	Sutterella	Rikenella	-1	Negative
Ruminococcus	Oscillospira	-1	Negative	Sutterella	Oscillospira	1	Positive
Ruminococcus	Rikenella	-1	Negative	Akkermansia	Prevotella	-1	Negative
Bacteroides	Prevotella	-1	Negative	Akkermansia	Sutterella	-1	Negative
Bacteroides	Ruminococcus	-1	Negative	Akkermansia	Rikenella	1	Positive
Bacteroides	Rikenella	0.6	Positive	Akkermansia	Lachnospira	-0.6	Negative
Bacteroides	Odoribacter	-0.5	Negative	Akkermansia	Oscillospira	-0.6	Negative
Bacteroides	Lachnospira	1	Positive	Lachnospira	Akkermansia	-0.6	Negative
Bacteroides	Akkermansia	-0.3	Negative	Lachnospira	Prevotella	1	Positive
Akkermansia	Bacteroides	-0.3	Negative	Lachnospira	Sutterella	-0.6	Negative
Akkermansia	Ruminococcus	0.5	Positive	Lachnospira	Oscillospira	-1	Negative
Akkermansia	Prevotella	-0.3	Negative	Oscillospira	Lachnospira	-1	Negative
Akkermansia	Odoribacter	-0.3	Negative	Oscillospira	Prevotella	1	Positive
Akkermansia	Rikenella	-0.5	Negative	Oscillospira	Sutterella	-0.6	Negative
Akkermansia	Lachnospira	1	Positive	Oscillospira	Akkermansia	-0.6	Negative
Lachnospira	Akkermansia	1	Positive	Oscillospira	Rikenella	-1	Negative
Lachnospira	Rikenella	-1	Negative	Rikenella	Oscillospira	-1	Negative
Lachnospira	Prevotella	-1	Negative	Rikenella	Prevotella	-1	Negative
Lachnospira	Bacteroides	1	Positive	Rikenella	Sutterella	-1	Negative

Lachnospira	Odoribacter	0.6	Positive	Rikenella	Lachnospira	-1	Negative
Odoribacter	Lachnospira	0.6	Positive	Rikenella	Akkermansia	1	Positive
Odoribacter	Akkermansia	-0.3	Negative				
Odoribacter	Bacteroides	-0.5	Negative				
Odoribacter	Ruminococcus	-0.5	Negative				
Odoribacter	Rikenella	1	Positive				
Odoribacter	Prevotella	-0.3	Negative				
Rikenella	Odoribacter	1	Positive				
Rikenella	Lachnospira	-1	Negative				
Rikenella	Bacteroides	0.6	Positive				
Rikenella	Ruminococcus	-0.5	Negative				
Rikenella	Prevotella	0.6	Positive				
Rikenella	Akkermansia	-0.3	Negative				
Oscillospira	Ruminococcus	-1	Negative				
**Aging Control (Phylum)**				**Aging Probiotic (Phylum)**			
Firmicutes	Bacteroidetes	1	Positive	Firmicutes	Actinobacteria	1	Positive
Firmicutes	Cyanobacteria	-0.3	Negative	Firmicutes	Proteobacteria	-0.5	Negative
Bacteroidetes	Cyanobacteria	-0.3	Negative	Firmicutes	Verrucomicrobia	-0.2	Negative
Bacteroidetes	Firmicutes	1	Positive	Firmicutes	Bacteroidetes	-1	Negative
Cyanobacteria	Firmicutes	-0.3	Negative	Bacteroidetes	Firmicutes	-1	Negative
Cyanobacteria	Bacteroidetes	-0.3	Negative	Bacteroidetes	Proteobacteria	0.5	Positive
Cyanobacteria	Proteobacteria	-0.5	Negative	Bacteroidetes	Verrucomicrobia	0.6	Positive
Cyanobacteria	Verrucomicrobia	-0.1	Negative	Bacteroidetes	Actinobacteria	-0.2	Negative
Verrucomicrobia	Cyanobacteria	-0.1	Negative	Proteobacteria	Actinobacteria	-0.2	Negative
Verrucomicrobia	Proteobacteria	-0.1	Negative	Proteobacteria	Firmicutes	-0.5	Negative
Proteobacteria	Verrucomicrobia	-0.1	Negative	Proteobacteria	Bacteroidetes	0.5	Positive
Proteobacteria	Cyanobacteria	-0.5	Negative	Proteobacteria	Verrucomicrobia	0.6	Positive

				Verrucomicrobia	Proteobacteria	0.6	Positive
				Verrucomicrobia	Firmicutes	-0.2	Negative
				Verrucomicrobia	Bacteroidetes	0.6	Positive
				Verrucomicrobia	Cyanobacteria	0.5	Positive
				Actinobacteria	Bacteroidetes	-0.2	Negative
				Actinobacteria	Proteobacteria	-0.2	Negative
				Actinobacteria	Firmicutes	1	Positive
				Cyanobacteria	Verrucomicrobia	0.5	Positive
**Aging Control (Genus)**				**Aging Probiotic (Genus)**			
**Taxon1**	**Taxon2**	**Correlation (p<0.05)**	**Association**	**Taxon1**	**Taxon2**	**Correlation (p<0.05)**	**Association**
Prevotella	Sutterella	1	Positive	Prevotella	Akkermansia	0.6	Positive
Prevotella	Bacteroides	-1	Negative	Prevotella	Oscillospira	-1	Negative
Prevotella	Ruminococcus	0.6	Positive	Prevotella	Ruminococcus	-0.6	Negative
Prevotella	Lactobacillus	-1	Negative	Prevotella	Lactobacillus	-1	Negative
Prevotella	Lachnospira	-0.4	Positive	Prevotella	Lachnospira	-0.5	Negative
Prevotella	Rikenella	1	Positive	Prevotella	Rikenella	-0.6	Negative
Bacteroides	Prevotella	-1	Negative	Akkermansia	Prevotella	0.6	Positive
Bacteroides	Ruminococcus	-0.5	Negative	Akkermansia	Oscillospira	-1	Negative
Bacteroides	Lactobacillus	1	Positive	Akkermansia	Ruminococcus	-0.3	Negative
Bacteroides	Lachnospira	0.6	Positive	Akkermansia	Lactobacillus	-1	Negative
Bacteroides	Sutterella	0.5	Positive	Akkermansia	Lachnospira	-1	Negative
Bacteroides	Rikenella	-1	Negative	Akkermansia	Rikenella	-1	Negative
Lactobacillus	Bacteroides	1	Positive	Ruminococcus	Lactobacillus	1	Positive
Lactobacillus	Prevotella	-1	Negative	Ruminococcus	Lachnospira	1	Positive
Lactobacillus	Lachnospira	-0.5	Negative	Ruminococcus	Rikenella	0.6	Positive
Lactobacillus	Sutterella	-0.3	Negative	Ruminococcus	Prevotella	-0.6	Negative
Lactobacillus	Ruminococcus	0.6	Positive	Ruminococcus	Oscillospira	1	Positive
Sutterella	Lactobacillus	-0.3	Negative	Ruminococcus	Prevotella	-0.6	Negative
Sutterella	Bacteroides	0.5	Positive	Ruminococcus	Akkermansia	-0.6	Negative

Sutterella	Prevotella	1	Positive	Oscillospira	Ruminococcus	1	Positive
Sutterella	Lachnospira	-0.5	Negative	Oscillospira	Prevotella	-1	Negative
Sutterella	Rikenella	-1	Negative	Oscillospira	Akkermansia	-1	Negative
Sutterella	Ruminococcus	0.5	Positive	Oscillospira	Lactobacillus	1	Positive
Lachnospira	Sutterella	-0.5	Negative	Oscillospira	Lachnospira	1	Positive
Lachnospira	Lactobacillus	-0.5	Negative	Oscillospira	Rikenella	0.6	Positive
Lachnospira	Bacteroides	0.6	Positive	Rikenella	Oscillospira	0.6	Positive
Lachnospira	Prevotella	-0.4	Positive	Rikenella	Ruminococcus	0.6	Positive
Lachnospira	Ruminococcus	-0.6	Negative	Rikenella	Prevotella	-0.6	Negative
Ruminococcus	Lachnospira	-0.6	Negative	Rikenella	Akkermansia	-1	Negative
Ruminococcus	Lactobacillus	0.6	Positive	Rikenella	Lactobacillus	1	Positive
Ruminococcus	Bacteroides	-0.5	Negative	Rikenella	Lachnospira	1	Positive
Ruminococcus	Prevotella	0.6	Positive	Lactobacillus	Rikenella	1	Positive
Ruminococcus	Sutterella	0.5	Positive	Lactobacillus	Oscillospira	1	Positive
Rikenella	Sutterella	-1	Negative	Lactobacillus	Ruminococcus	1	Positive
Rikenella	Bacteroides	-1	Negative	Lactobacillus	Akkermansia	-1	Negative
Rikenella	Prevotella	1	Positive	Lactobacillus	Prevotella	-0.5	Positive
				Lactobacillus	Lachnospira	1	Positive
				Lachnospira	Lactobacillus	1	Positive
				Lachnospira	Rikenella	1	Positive
				Lachnospira	Oscillospira	1	Positive
				Lachnospira	Ruminococcus	1	Positive
				Lachnospira	Akkermansia	-1	Negative
				Lachnospira	Prevotella	-0.5	Positive

Table S4: Summary of cecal microbiome co-occurrences between young and aging mice at phylum and genus levels based on Kendall's Tau correlation analysis after which FDR (q<0.05) correction was performed.

Young Control (Phylum)				Young Probiotic (Phylum)			
**Taxon1**	**Taxon2**	**Correlation ($p<0.05$)**	**Association**	**Taxon1**	**Taxon2**	**Correlation ($p<0.05$)**	**Association**
Firmicutes	Bacteroidetes	-1	Negative	Firmicutes	Bacteroidetes	1	Positive
Firmicutes	Cyanobacteria	-0.5	Negative	Firmicutes	Cyanobacteria	-0.5	Negative
Firmicutes	Verrucomicrobia	-0.6	Negative	Firmicutes	Verrucomicrobia	0.6	Positive
Firmicutes	Proteobacteria	-0.3	Negative	Firmicutes	Actinobacteria	-0.3	Negative
Proteobacteria	Firmicutes	-0.3	Negative	Actinobacteria	Firmicutes	-0.3	Negative
Proteobacteria	Bacteroidetes	1	Positive	Actinobacteria	Bacteroidetes	-0.2	Negative
Proteobacteria	Cyanobacteria	0.6	Positive	Actinobacteria	Proteobacteria	-0.5	Negative
Cyanobacteria	Verrucomicrobia	0.5	Positive	Actinobacteria	Verrucomicrobia	-0.4	Negative
Cyanobacteria	Proteobacteria	0.4	Positive	Verrucomicrobia	Actinobacteria	-0.4	Negative
Cyanobacteria	Firmicutes	-0.5	Negative	Verrucomicrobia	Firmicutes	0.3	Positive
Cyanobacteria	Bacteroidetes	1	Positive	Verrucomicrobia	Bacteroidetes	1	Positive
Bacteroidetes	Cyanobacteria	1	Positive	Verrucomicrobia	Cyanobacteria	-1	Negative
Bacteroidetes	Proteobacteria	1	Positive	Verrucomicrobia	Proteobacteria	-0.4	Negative
Bacteroidetes	Firmicutes	-1	Negative	Proteobacteria	Verrucomicrobia	-0.6	Negative
Bacteroidetes	Verrucomicrobia	-0.5	Negative	Proteobacteria	Actinobacteria	-0.5	Negative
Verrucomicrobia	Bacteroidetes	-0.5	Negative	Proteobacteria	Cyanobacteria	-0.3	Negative
Verrucomicrobia	Firmicutes	-0.6	Negative	Cyanobacteria	Proteobacteria	-0.3	Negative
Verrucomicrobia	Cyanobacteria	0.5	Positive	Cyanobacteria	Firmicutes	-0.3	Negative
				Cyanobacteria	Verrucomicrobia	-1	Negative
				Cyanobacteria	Bacteroidetes	1	Positive

				Bacteroidetes	Cyanobacteria	-1	Negative
				Bacteroidetes	Verrucomicrobia	-1	Negative
				Bacteroidetes	Actinobacteria	-0.2	Negative
				Bacteroidetes	Firmicutes	0.3	Positive
**Young Control (Genus)**				**Young Probiotic (Genus)**			
**Taxon1**	**Taxon2**	**Correlation ($p<0.05$)**	**Association**	**Taxon1**	**Taxon2**	**Correlation ($p<0.05$)**	**Association**
Prevotella	Oscillospira	0.6	Positive	Prevotella	Oscillospira	0.6	Positive
Prevotella	Akkermansia	-0.5	Negative	Prevotella	Akkermansia	-0.5	Negative
Prevotella	Lachnospira	-1	Negative	Prevotella	Lachnospira	1	Positive
Prevotella	Sutterella	-0.4	Negative	Prevotella	Sutterella	0.4	Positive
Prevotella	Bacteroides	-1	Negative	Prevotella	Lactobacillus	0.4	Positive
Bacteroides	Prevotella	-1	Negative	Prevotella	odoribacteria	0.6	Positive
Bacteroides	Oscillospira	-0.6	Negative	Prevotella	Rikenella	-1	Negative
Bacteroides	Rikenella	0.6	Positive	Prevotella	Ruminococcus	1	Positive
Bacteroides	Lachnospira	1	Positive	Prevotella	Bacteroides	1	Positive
Bacteroides	Sutterella	-0.3	Negative	Bacteroides	Prevotella	1	Positive
Bacteroides	Ruminococcus	-0.6	Negative	Bacteroides	Oscillospira	-0.5	Negative
Ruminococcus	Bacteroides	-0.6	Negative	Bacteroides	Akkermansia	-0.5	Negative
Ruminococcus	Akkermansia	0.5	Positive	Bacteroides	Lachnospira	1	Positive
Ruminococcus	Sutterella	0.6	Positive	Bacteroides	Sutterella	0.6	Positive
Ruminococcus	Lachnospira	-1	Negative	Bacteroides	Lactobacillus	0.4	Positive
Lachnospira	Ruminococcus	-1	Negative	Bacteroides	odoribacteria	-0.4	Negative
Lachnospira	Bacteroides	1	Positive	Bacteroides	Rikenella	-1	Negative
Lachnospira	Prevotella	-1	Negative	Rikenella	Bacteroides	-1	Negative
Lachnospira	Oscillospira	-0.6	Negative	Rikenella	Prevotella	-1	Negative
Oscillospira	Lachnospira	-0.6	Negative	Rikenella	Oscillospira	-0.4	Negative
Oscillospira	Prevotella	0.6	Positive	Rikenella	Odoribacteria	-0.5	Negative
Oscillospira	Bacteroides	-0.6	Negative	Rikenella	Lachnospira	1	Positive
Oscillospira	Sutterella	-0.3	Negative	Rikenella	Sutterella	0.5	Positive

Oscillospira	Akkermansia	-0.6	Negative	Rikenella	Ruminococcus	-0.6	Negative
Akkermansia	Oscillospira	-0.6	Negative	Rikenella	Lactobacillus	-0.4	Negative
Akkermansia	Ruminococcus	0.5	Positive	Lactobacillus	Rikenella	-0.4	Negative
Akkermansia	Prevotella	-0.5	Negative	Lactobacillus	Prevotella	0.4	Positive
Akkermansia	Sutterella	0.6	Positive	Lactobacillus	Bacteroides	0.4	Positive
Sutterella	Akkermansia	0.6	Positive	Lactobacillus	Ruminococcus	0.6	Positive
Sutterella	Oscillospira	-0.3	Negative	Lactobacillus	Akkermansia	-0.5	Negative
Sutterella	Ruminococcus	0.6	Positive	Lactobacillus	Lachnospira	-1	Negative
Sutterella	Bacteroides	-0.3	Negative	Lactobacillus	Odoribacteria	-0.6	Negative
				Lactobacillus	Oscillospira	0.4	Positive
				Lactobacillus	Sutterella	0.5	Positive
Sutterella	Prevotella	-0.4	Negative	Odoribacteria	Lactobacillus	-0.6	Negative
Sutterella	Rikenella	-0.6	Negative	Odoribacteria	Ruminococcus	0.6	Positive
Rikenella	Sutterella	-0.6	Negative	Odoribacteria	Rikenella	-0.5	Negative
Rikenella	Bacteroides	0.6	Positive	Odoribacteria	Prevotella	0.6	Positive
				Odoribacteria	Bacteroides	-0.4	Negative
				Odoribacteria	Sutterella	-0.5	Negative
				Odoribacteria	Lachnospira	1	Positive
				Lachnospira	Odoribacteria	1	Positive
				Lachnospira	Rikenella	1	Positive
				Lachnospira	Prevotella	1	Positive
				Lachnospira	Bacteroides	1	Positive
				Lachnospira	Lactobacillus	-1	Negative
				Lachnospira	Oscillospira	1	Positive
				Lachnospira	Sutterella	0.6	Positive
				Lachnospira	Ruminococcus	0.6	Positive
				Ruminococcus	Lachnospira	0.6	Positive
				Ruminococcus	Odoribacteria	0.6	Positive
				Ruminococcus	Lactobacillus	0.6	Positive
				Ruminococcus	Rikenella	-0.6	Negative
				Ruminococcus	Prevotella	1	Positive

				Ruminococcus	Oscillospira	1	Positive
				Ruminococcus	Akkermansia	-1	Negative
				Akkermansia	Ruminococcus	-1	Negative
				Akkermansia	Prevotella	-0.5	Negative
				Akkermansia	Suterella	-0.5	Negative
				Akkermansia	Lactobacillus	-0.5	Negative
				Akkermansia	Bacteroides	-0.5	Negative
				Akkermansia	Oscillospira	1	Positive
				Oscillospira	Akkermansia	1	Positive
				Oscillospira	Prevotella	0.6	Positive
				Oscillospira	Bacteroides	-0.5	Negative
				Oscillospira	Rikenella	-0.4	Negative
				Oscillospira	Lactobacillus	0.4	Positive
				Oscillospira	Lachnospira	1	Positive
				Oscillospira	Ruminococcus	1	Positive
				Oscillospira	Akkermansia	1	Positive
				Oscillospira	Sutterella	-0.5	Negative
				Sutterella	Oscillospira	-0.5	Negative
				Sutterella	Akkermansia	-0.5	Negative
				Sutterella	Lachnospira	0.6	Positive
				Sutterella	Rikenella	0.5	Positive
				Sutterella	Bacteroides	0.6	Positive
				Sutterella	Prevotella	0.4	Positive
				Sutterella	Odoribacteria	-0.5	Negative
				Sutterella	Lactobacillus	0.5	Positive
**Aging Control (Phylum)**				**Aging Probiotic (Phylum)**			
**Taxon1**	**Taxon2**	**Correlation (p<0.05)**	**Association**	**Taxon1**	**Taxon2**	**Correlation (p<0.05)**	**Association**
Firmicutes	Actinobacteria	1	Positive	Firmicutes	Bacteroidetes	1	Positive
Firmicutes	Bacteroidetes	-1	Negative	Firmicutes	Proteobacteria	0.6	Positive

Firmicutes	Verrucomicrobia	-0.3	Negative	Firmicutes	Cyanobacteria	-0.5	Negative
Bacteroidetes	Firmicutes	-1	Negative	Firmicutes	Verrucomicrobi a	0.6	Positive
Bacteroidetes	Actinobacteria	-0.3	Negative	Firmicutes	Actinobacteria	-0.3	Negative
Bacteroidetes	Verrucomicrobia	0.5	Positive	Bacteroidetes	Firmicutes	1	Positive
Verrucomicrobi a	Bacteroidetes	0.5	Positive	Bacteroidetes	Proteobacteria	0.5	Positive
Verrucomicrobi a	Firmicutes	-0.3	Negative	Bacteroidetes	Cyanobacteria	-0.5	Negative
Verrucomicrobi a	Proteobacteria	0.6	Positive	Bacteroidetes	Verrucomicrobi a	0.6	Positive
Verrucomicrobi a	Actinobacteria	-0.5	Negative	Bacteroidetes	Actinobacteria	-0.2	Negative
Actinobacteria	Verrucomicrobia	-0.5	Negative	Actinobacteria	Bacteroidetes	-0.2	Negative
Actinobacteria	Firmicutes	1	Positive	Actinobacteria	Firmicutes	-0.3	Negative
Actinobacteria	Bacteroidetes	-0.3	Negative	Actinobacteria	Proteobacteria	-0.5	Negative
Actinobacteria	Proteobacteria	-0.3	Negative	Actinobacteria	Cyanobacteria	-0.5	Negative
Proteobacteria	Actinobacteria	-0.3	Negative	Actinobacteria	Verrucomicrobi a	-0.6	Negative
Proteobacteria	Verrucomicrobia	0.6	Positive	Verrucomicrobia	Actinobacteria	-0.6	Negative
				Verrucomicrobia	Bacteroidetes	0.6	Positive
				Verrucomicrobia	Firmicutes	0.6	Positive
				Verrucomicrobia	Proteobacteria	-0.5	Negative
				Verrucomicrobia	Cyanobacteria	-0.5	Negative
				Cyanobacteria	Verrucomicrobi a	-0.5	Negative
				Cyanobacteria	Actinobacteria	-0.5	Negative
				Cyanobacteria	Bacteroidetes	-0.5	Negative
				Cyanobacteria	Firmicutes	-0.5	Negative
				Cyanobacteria	Proteobacteria	-0.3	Negative
				Proteobacteria	Cyanobacteria	-0.3	Negative
				Proteobacteria	Verrucomicrobi a	-0.5	Negative

				Proteobacteria	Actinobacteria	-0.5	Negative
				Proteobacteria	Bacteroidetes	0.5	Positive
				Proteobacteria	Firmicutes	0.6	Positive
**Aging Control (Genus)**				**Aging Probiotic (Genus)**			
**Taxon1**	**Taxon2**	**Correlation (p<0.05)**	**Association**	**Taxon1**	**Taxon2**	**Correlation (p<0.05)**	**Association**
Prevotella	Rikenella	1	Positive	Prevotella	Rikenella	-1	Negative
Prevotella	Sutterella	-0.6	Negative	Prevotella	Sutterella	-0.6	Negative
Prevotella	Lachnospira	-1	Negative	Prevotella	Lachnospira	1	Positive
Prevotella	Oscillospira	-1	Negative	Prevotella	Oscillospira	1	Positive
Prevotella	Bacteroides	-0.5	Negative	Prevotella	Bacteroides	-0.5	Negative
Bacteroides	Prevotella	-0.5	Negative	Prevotella	Ruminococcus	-1	Negative
Bacteroides	Sutterella	1	Positive	Prevotella	Lactobacillus	1	Positive
Bacteroides	Oscillospira	-1	Negative	Lactobacillus	Prevotella	1	Positive
Bacteroides	Lachnospira	1	Positive	Lactobacillus	Rikenella	-0.6	Negative
Lachnospira	Bacteroides	1	Positive	Lactobacillus	Sutterella	-0.4	Negative
Lachnospira	Sutterella	-0.6	Negative	Lactobacillus	Dorea	-0.8	Negative
Lachnospira	Prevotella	-1	Negative	Lactobacillus	Oscillospira	0.6	Positive
Lachnospira	Rikenella	1	Positive	Lactobacillus	Bacteroides	-0.5	Negative
Lachnospira	Oscillospira	1	Positive	Lactobacillus	Ruminococcus	-1	Negative
Oscillospira	Lachnospira	1	Positive	Ruminococcus	Lactobacillus	-1	Negative
Oscillospira	Prevotella	-1	Negative	Ruminococcus	Prevotella	-1	Negative
Oscillospira	Bacteroides	-1	Negative	Ruminococcus	Rikenella	0.5	Positive
Oscillospira	Sutterella	1	Positive	Ruminococcus	Sutterella	-0.3	Negative
Oscillospira	Rikenella	-1	Negative	Ruminococcus	Dorea	0.6	Positive
Rikenella	Oscillospira	-1	Negative	Ruminococcus	Oscillospira	-0.5	Negative
Rikenella	Prevotella	1	Positive	Ruminococcus	Bacteroides	0.6	Positive
Rikenella	Lachnospira	1	Positive	Ruminococcus	Lachnospira	0.8	Positive
Rikenella	Sutterella	-0.6	Negative	Lachnospira	Ruminococcus	0.8	Positive
Sutterella	Rikenella	-0.6	Negative	Lachnospira	Bacteroides	-0.8	Negative

Sutterella	Oscillospira	1	Positive	Lachnospira	Prevotella	1	Positive
Sutterella	Prevotella	-0.6	Negative	Lachnospira	Rikenella	0.6	Positive
Sutterella	Lachnospira	-0.6	Negative	Lachnospira	Sutterella	-0.6	Negative
Sutterella	Bacteroides	1	Positive	Lachnospira	Dorea	-0.8	Negative
				Lachnospira	Oscillospira	-1	Negative
				Oscillospira	Lachnospira	-1	Negative
				Oscillospira	Prevotella	1	Positive
				Oscillospira	Lactobacillus	0.6	Positive
				Oscillospira	Ruminococcus	-0.5	Negative
				Oscillospira	Dorea	-0.4	Negative
				Oscillospira	Sutterella	-0.8	Negative
				Oscillospira	Rikenella	-1	Negative
				Oscillospira	Bacteroides	-0.6	Negative
				Bacteroides	Oscillospira	-0.6	Negative
				Bacteroides	Prevotella	-0.5	Negative
				Bacteroides	Lactobacillus	-0.5	Negative
				Bacteroides	Ruminococcus	0.6	Positive
				Bacteroides	Lachnospira	-0.8	Negative
				Bacteroides	Dorea	0.8	Positive
				Bacteroides	Sutterella	0.8	Positive
				Bacteroides	Rikenella	0.6	Positive
				Rikenella	Bacteroides	0.6	Positive
				Rikenella	Oscillospira	-1	Negative
				Rikenella	Prevotella	-1	Negative
				Rikenella	Lactobacillus	-0.6	Negative
				Rikenella	Ruminococcus	0.5	Positive
				Rikenella	Lachnospira	0.6	Positive
				Rikenella	Dorea	0.6	Positive
				Rikenella	Sutterella	0.8	Positive
				Sutterella	Rikenella	0.8	Positive
				Sutterella	Bacteroides	0.8	Positive
				Sutterella	Oscillospira	-0.8	Negative

				Sutterella	Prevotella	-0.6	Negative
				Sutterella	Lactobacillus	-0.4	Negative
				Sutterella	Ruminococcus	-0.3	Negative
				Sutterella	Lachnospira	-0.6	Negative
				Sutterella	Dorea	0.6	Positive
				Dorea	Sutterella	0.6	Positive
				Dorea	Rikenella	0.6	Positive
				Dorea	Bacteroides	0.8	Positive
				Dorea	Oscillospira	-0.4	Negative
				Dorea	Lachnospira	-0.8	Negative
				Dorea	Lactobacillus	-0.8	Negative
				Dorea	Ruminococcus	0.6	Positive

Figure S1: Bacterial taxa identified to be differentially abundant by linear discrimination analysis (LDA) effect size (LEfSe, log LDA > 2.0) analysis in all the 4 samples at phylum level (**A**) and genus level (**B**). The values are shown as the mean ± SEM. *Significant differences with p <0.05.

A

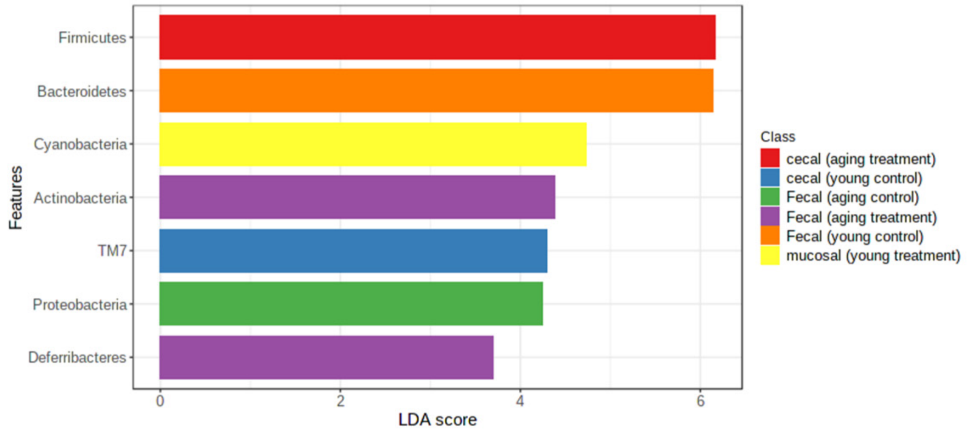

B

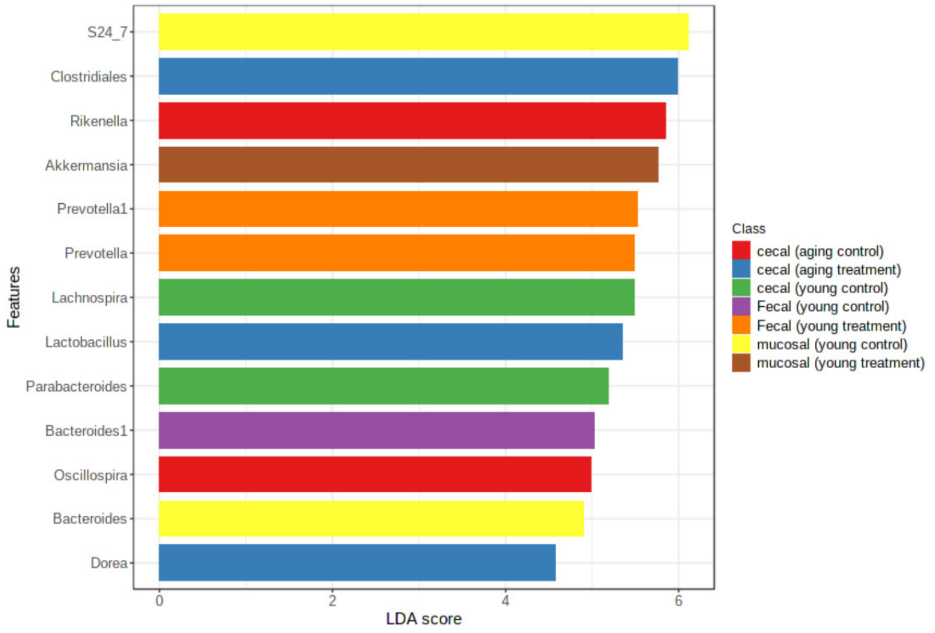

Figure S2. Summary of co-occurrences and co-exclusion analysis between young and aging mice at the phylum and genus levels in fecal (**A-B**) and mucosal (**C-D**) cecal (**E-F**). (YC) Young control group, (YP) young probiotic group, (AC) aging control group and (AP) aging probiotic group.

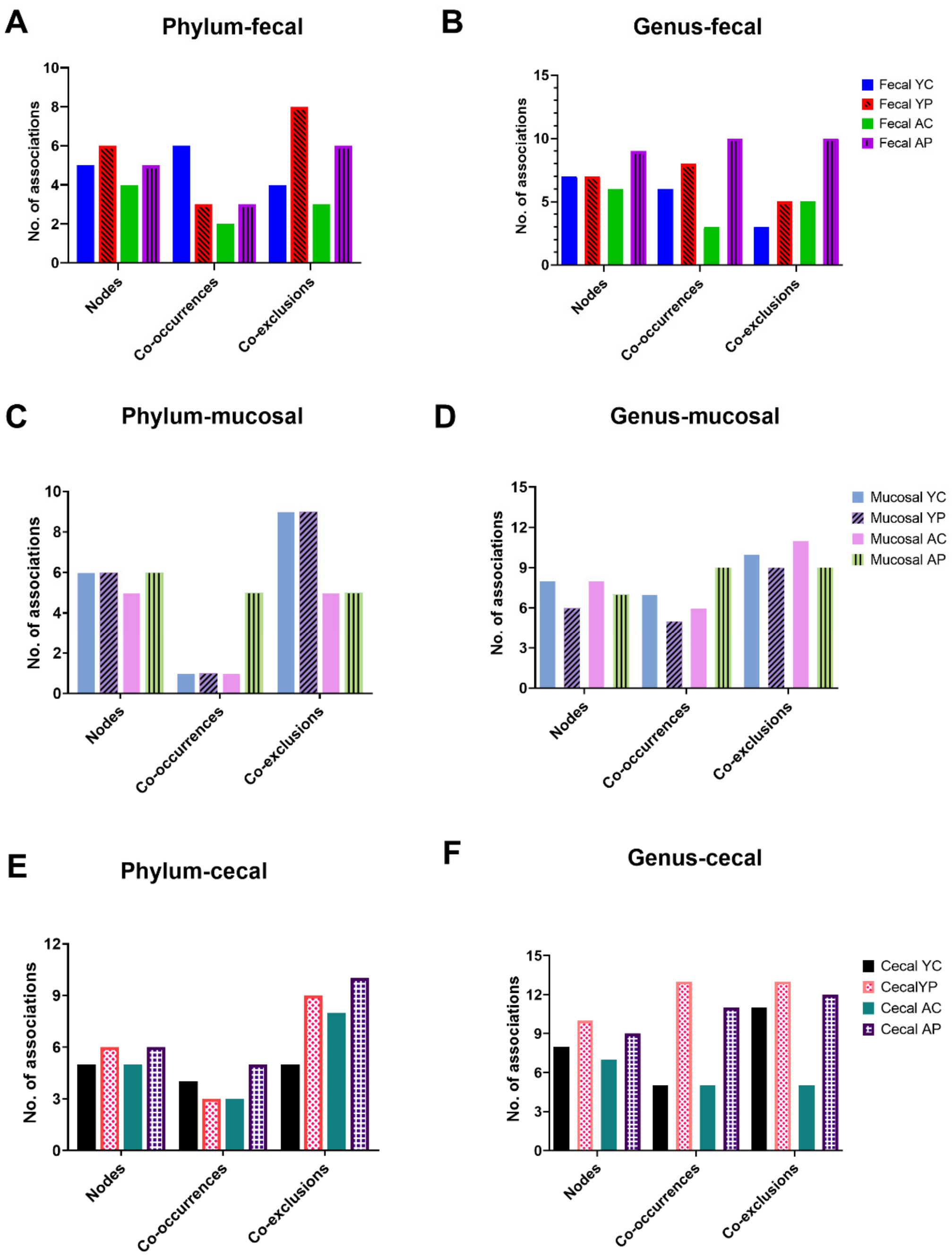

Supplement: Supplementary file 1 [file nutrients-14-00977-s001.zip › nutrients-1611982-supplementary.pdf]
